# What should researchers do in the era of artificial intelligence?

**DOI:** 10.12701/jyms.2026.43.2

**Published:** 2025-12-15

**Authors:** Min Cheol Chang

**Affiliations:** Department of Physical Medicine and Rehabilitation, Yeungnam University College of Medicine, Daegu, Korea


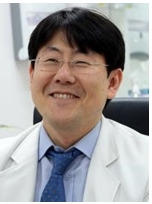


The current pace of change in medical research has surpassed that of previous technological turning points. Emerging large language models are transforming how research is performed and papers are written, redefining the roles of researchers [1-4].

## From the era of searching and reading papers to the era of asking artificial intelligence

Previously, researchers were required to read and summarize numerous research papers and reviews. However, nowadays, researchers can instantly obtain the outline and core concepts of papers with reasonable accuracy by asking artificial intelligence (AI) tools such as Generative Pre-trained Transformer (GPT) to “Summarize recent research trends on this topic.” AI is no longer a simple research tool but has established itself as a knowledge partner that integrates, structures, and provides the information that researchers require. Although AI’s answers are not yet perfect, it is evident that how we read and review papers before starting research is changing to “asking AI first to set the research direction.” Moreover, when clinicians previously had insufficient knowledge during the process of diagnosing and treating patients, they searched for and read related papers, integrated and summarized them, and then applied them in clinical practice. However, they can now directly ask AI tools, such as GPT, what they do not know and can make clinical judgments based on the answers.

## The era of artificial intelligence-assisted paper writing: the changing roles of researchers

An increasing number of researchers are actively using AI to write abstracts, introductions, methods, and discussions. The first draft can be generated when they instruct AI to “write in this order, including these contents, and in this tone.” AI now offers increasingly advanced levels of automation, not only for sentence construction, but also for logical structures, reference formatting, tables, and figures. This implies that “designing the structure and content of manuscripts” is an emerging and important capability. In other words, researchers are becoming supervisors and evaluators rather than primary producers of sentences.

## Core abilities of the future: “ability to discern what to research” and “ability to directly create research data”

Although AI can summarize the existing literature, highlight knowledge gaps, and suggest potential research directions, it cannot fully replace the deeper contextual judgment required to determine which ideas are truly meaningful, clinically relevant, or feasible. The ability to discern what should be studied, which is grounded in real-world experience, intuition, and domain expertise, remains a unique human capacity. Furthermore, AI cannot independently generate real patient data or conduct experiments. Thus, for clinicians and researchers, the ability to accumulate patient data, conduct experiments, and create data is a key competitive advantage.

## Conclusion: artificial intelligence redefines the role of researchers

AI saves researchers time, increases knowledge accessibility, and maximizes research efficiency. Although AI can summarize existing research, highlight knowledge gaps, and even suggest potential directions for future studies, deeper contextual evaluations, such as determining clinical relevance, prioritizing meaningful research questions, and interpreting findings in real-world settings, still require human expertise. Moreover, AI cannot independently generate clinical or experimental data, making the ability to directly create and accumulate research data an essential competency for future researchers. As AI continues to evolve, researchers increasingly function as “designers of knowledge” who set research directions, ensure scientific validity, and produce data based on their own insights. However, despite its growing utility, AI has some limitations, including the risk of hallucinations, ethical concerns such as potential patient data exposure, and the need for greater transparency in the production and use of AI-generated information. The development of AI is a transformative change that redefines researchers’ roles and requires them to assume more advanced responsibilities. We are already standing at the center of these changes.
